# A Chromosome-Length Reference Genome for the Endangered Pacific Pocket Mouse Reveals Recent Inbreeding in a Historically Large Population

**DOI:** 10.1093/gbe/evac122

**Published:** 2022-07-27

**Authors:** Aryn P Wilder, Olga Dudchenko, Caitlin Curry, Marisa Korody, Sheela P Turbek, Mark Daly, Ann Misuraca, Gaojianyong Wang, Ruqayya Khan, David Weisz, Julie Fronczek, Erez Lieberman Aiden, Marlys L Houck, Debra M Shier, Oliver A Ryder, Cynthia C Steiner

**Affiliations:** Conservation Science Wildlife Health, San Diego Zoo Wildlife Alliance, Escondido, CA, USA; The Center for Genome Architecture, Department of Molecular and Human Genetics, Baylor College of Medicine, Houston, TX, USA; Center for Theoretical Biological Physics and Department of Computer Science, Rice University, Houston, TX, USA; Conservation Science Wildlife Health, San Diego Zoo Wildlife Alliance, Escondido, CA, USA; Conservation Science Wildlife Health, San Diego Zoo Wildlife Alliance, Escondido, CA, USA; Conservation Science Wildlife Health, San Diego Zoo Wildlife Alliance, Escondido, CA, USA; Ecology and Evolutionary Biology, University of Colorado, Boulder, CO, USA; Dovetail Genomics, Scotts Valley, CA, USA; Conservation Science Wildlife Health, San Diego Zoo Wildlife Alliance, Escondido, CA, USA; Department of Genome Regulation, Max Planck Institute for Molecular Genetics, Berlin, Germany; The Center for Genome Architecture, Department of Molecular and Human Genetics, Baylor College of Medicine, Houston, TX, USA; The Center for Genome Architecture, Department of Molecular and Human Genetics, Baylor College of Medicine, Houston, TX, USA; Conservation Science Wildlife Health, San Diego Zoo Wildlife Alliance, Escondido, CA, USA; The Center for Genome Architecture, Department of Molecular and Human Genetics, Baylor College of Medicine, Houston, TX, USA; Center for Theoretical Biological Physics and Department of Computer Science, Rice University, Houston, TX, USA; UWA School of Agriculture and Environment, The University of Western Australia, Crawley, Australia; Broad Institute of MIT and Harvard, Cambridge, MA, USA; Shanghai Institute for Advanced Immunochemical Studies, ShanghaiTech, China; Conservation Science Wildlife Health, San Diego Zoo Wildlife Alliance, Escondido, CA, USA; Conservation Science Wildlife Health, San Diego Zoo Wildlife Alliance, Escondido, CA, USA; Department of Ecology & Evolutionary Biology, University of California Los Angeles, Los Angeles, CA, USA; Conservation Science Wildlife Health, San Diego Zoo Wildlife Alliance, Escondido, CA, USA; Conservation Science Wildlife Health, San Diego Zoo Wildlife Alliance, Escondido, CA, USA

**Keywords:** runs of homozygosity, effective population size, HiFi, chromosome conformation capture, mitochondrial genome

## Abstract

High-quality reference genomes are fundamental tools for understanding population history, and can provide estimates of genetic and demographic parameters relevant to the conservation of biodiversity. The federally endangered Pacific pocket mouse (PPM), which persists in three small, isolated populations in southern California, is a promising model for studying how demographic history shapes genetic diversity, and how diversity in turn may influence extinction risk. To facilitate these studies in PPM, we combined PacBio HiFi long reads with Omni-C and Hi-C data to generate a de novo genome assembly, and annotated the genome using RNAseq. The assembly comprised 28 chromosome-length scaffolds (N50 = 72.6 MB) and the complete mitochondrial genome, and included a long heterochromatic region on chromosome 18 not represented in the previously available short-read assembly. Heterozygosity was highly variable across the genome of the reference individual, with 18% of windows falling in runs of homozygosity (ROH) >1 MB, and nearly 9% in tracts spanning >5 MB. Yet outside of ROH, heterozygosity was relatively high (0.0027), and historical *N_e_* estimates were large. These patterns of genetic variation suggest recent inbreeding in a formerly large population. Currently the most contiguous assembly for a heteromyid rodent, this reference genome provides insight into the past and recent demographic history of the population, and will be a critical tool for management and future studies of outbreeding depression, inbreeding depression, and genetic load.

**Significance**

The design of optimal conservation strategies for the federally endangered Pacific pocket mouse, a subspecies that has undergone substantial declines and is under intensive management, relies on precise estimates of genome-wide heterozygosity, inbreeding, demographic history, and chromosomal variation. We generated a chromosome-length reference genome for this species, and found massive stretches of homozygosity across a genome with relatively high baseline heterozygosity, reflecting inbreeding in a formerly large population. The high degree of contiguity of this reference genome enabled the detection of signatures of contemporary inbreeding, highlighting the value of high-quality reference genomes for the management of endangered species, and conservation of biodiversity.

## Introduction

Chromosome-length reference genomes are fundamental tools for addressing questions in biology, from evolutionary innovation to the underpinnings of disease (Rhie *et al*. 2021; Blaxter *et al*. 2022). Highly contiguous assemblies provide a more complete understanding of historical and contemporary diversity and demographics, and can enable the precise estimation of parameters relevant for the study, management, and conservation of endangered species (Robinson *et al*. 2021; Totikov *et al*. 2021).

The federally endangered Pacific pocket mouse (PPM; *Perognathus longimembris pacificus*) is a valuable model species for studying the interplay between genetic variation and extinction risk. Having been extirpated from most of its distribution along coastal southern California in the 1930s (Brylski *et al*. 1998; King *et al*. 2022), the subspecies persists in three small, isolated populations in Orange and San Diego counties with very low effective population sizes (*N*_e_ = 3.3, 25.0, and 50.6; Swei *et al*. 2003; Wilder *et al*. 2020). It is the target of intensive conservation measures involving both in situ management of wild populations and an ex situ breeding program for reintroduction (Brylski *et al*. 1998; King *et al*. 2022). Microsatellite and mitochondrial markers have provided estimates of population differentiation, diversity, *N_e_*, and relatedness, but genomic data would give additional resolution needed to address remaining questions relevant to population management. For example, a fitness analysis suggested that inbreeding and genetic load may play a role in limiting reproductive success (Wilder *et al*. 2020), and evidence of karyotype variation within *P. longimembris* (Patton 1967; McKnight and Lee 1992) and within PPM (King *et al*. 2020) has raised concerns that outbreeding depression could constrain fitness when populations are crossed. The use of genomic data to address these questions has been hindered by the lack of a highly contiguous reference genome. A draft genome for PPM, generated as part of a multispecies alignment of 241 mammalian genomes, was previously assembled from short-read sequence data using DISCOVAR (Zoonomia Consortium 2020). However, this genome was too fragmented (scaffold N50 < 25 KB) and incomplete (71.7% complete BUSCO genes; table 1) to study structural variation that may contribute to outbreeding depression, or to estimate long runs of homozygosity (ROH) to infer inbreeding coefficients.

**Table 1:** Genome Assembly Statistics Comparing the Final Assembly (HiFi + Omni-C + Hi-C) to the Previous DISCOVAR Assembly

	Final Assembly	DISCOVAR Assembly
Number of scaffolds	6,180	2,409,818
Total size of scaffolds	2,212,099,196	2,601,695,796
Longest scaffold	163,161,067	625,731
N50 scaffold length	72,679,016	24,714
L50 scaffold count	11	23,202
N50 contig length	7,389,774	17,686
L50 contig count	73	34,664
GC content	41.98%	41.84%
Complete BUSCOs	246 (96.4%)	183 (71.7%)
Complete & Single-Copy BUSCOs	239 (93.7%)	175 (68.6%)
Complete & Duplicated BUSCOs	7 (2.7%)	8 (3.1%)
Fragmented BUSCOs	3 (1.2%)	46 (18.0%)
Missing BUSCOs	6 (2.4%)	26 (10.3%)

Here, we combined PacBio HiFi long reads with both Dovetail Omni-C and Hi-C data to assemble a reference genome with chromosome-length scaffolds and a complete mitochondrial genome. We then mapped short-read data to the genome to estimate demographic history, heterozygosity, and ROH across the genome of the reference individual.

## Results and Discussion

### De Novo Genome Assembly

**Fig. 1.**
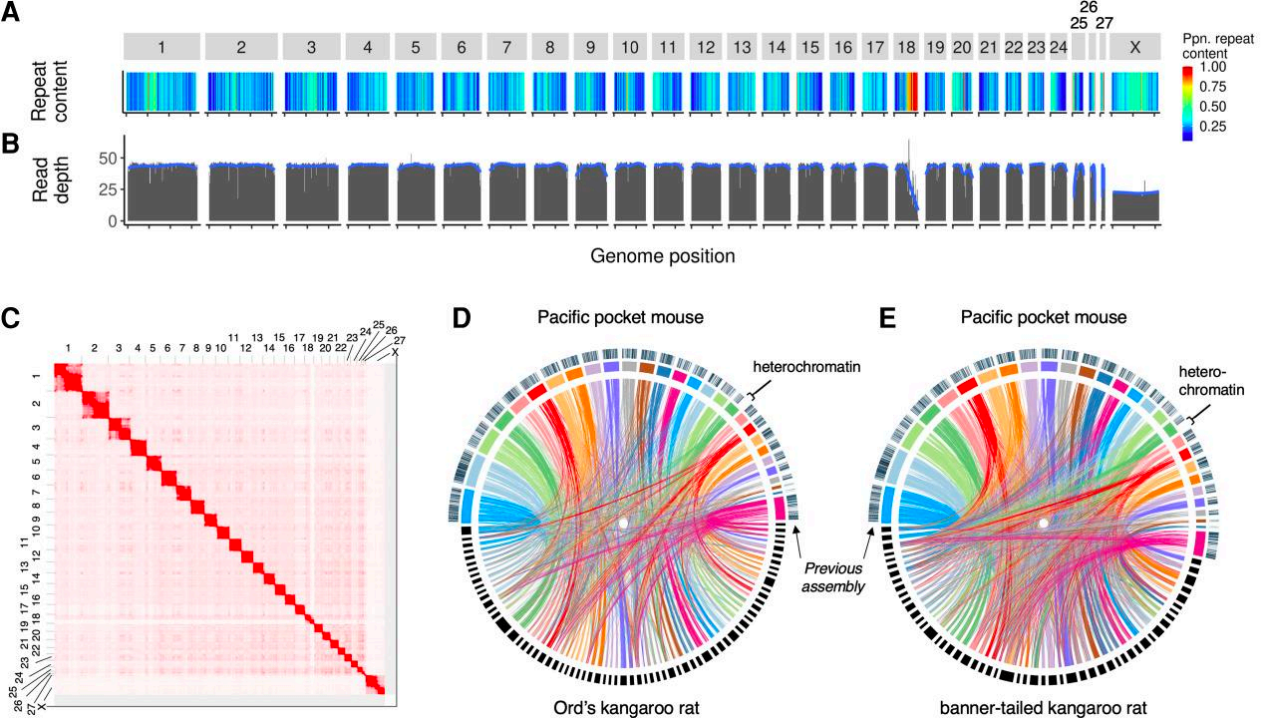
Chromosome-length scaffolds of the PPM genome assembled from HiFi, Omni-C, and Hi-C data. (*A*) Repeat content in 500 KB windows. Warmer colors show higher repeat content. (*B*) Mapping depth of short reads from the same individual. (*C*) Heatmap showing, on the scale from white to red, the frequency of 3D contact between any two loci of the PPM genome as measured by Hi-C sequencing. The same Hi-C data were used for scaffolding the genome to chromosome-length. Twenty-eight squares along the diagonal represent chromosome territories. An interactive version of this figure is available at https://tinyurl.com/25vywofz (Robinson *et al*. 2018). (*D* and *E*) Synteny between the PPM genome and two kangaroo rats, *D. ordii* (*D*) and *D. spectabilis* (*E*). Chromosome-length scaffolds of the PPM genome are colored, and scaffolds/contigs of the kangaroo rat genomes are black. Lines between the genomes show alignments >500 bp. Bars above the PPM scaffolds show the location of scaffolds >100 KB from the previous PPM genome assembly. The heterochromatic region of chromosome 18, absent from the larger scaffolds of the previous assembly, is highlighted.

The final genome assembly comprised 6,180 scaffolds (scaffold N50 = 72.68 MB), including 28 chromosome-length scaffolds (fig. 1; table 1). The number of chromosome-length scaffolds is in agreement with the inferred ancestral karyotype for *P. longimembris* (2*n* = 56; McKnight 1995). The anchored scaffold length comprises 89.6% of the total genome length (1,922,316,746 bp of 2,212,099,196 bp total), that is ∼290 Mb remain unanchored. Of this, ∼20 Mb are identifiable as Y-chromosome-related (based on elevated contact frequency with pseudoautosomal regions of the X-chromosome). The rest appears to be hard-to-assemble, repeat-rich heterochromatin, and centromeric repeats. The total assembly length of 2.21 GB was smaller than that of the previous draft DISCOVAR assembly (2.60 GB). Genome size estimated from kmers of publicly available short-read data generated for the draft assembly (SRR11431899) ranged from 1.86 to 1.94 GB (supplementary fig. S3 and table S6, Supplementary Material online), which is closer to the size of our assembly. The assembly had 96.4% complete BUSCO genes in the Eukaryota database (eukaryota_odb10), and 92.3% complete BUSCO genes in the Glires database (glires_odb10). The gene annotation for the nuclear genome had 93.5% complete genes in the glires_odb10 BUSCO database, with 0.6% fragmented genes and 2.6% missing genes. The complete mitochondrial genome was 16,293 bp (38.6% GC content), and included the control region, 22 tRNA genes, 2 ribosomal RNA genes, and 13 protein-coding genes (supplementary fig. S2 and table S3, Supplementary Material online).

Using RepeatMasker (Smit 2004), 41% of the genome was classified as repetitive, largely in the form of short- and long-interspersed nuclear elements and long terminal repeats (supplementary table S5, Supplementary Material online). Using de novo repeat identification (Girgis 2015), 29.6% of the genome was repetitive, including a >20 MB span of chromosome 18 (fig. 1*A*). This heterochromatic region was not represented among the larger contigs (>100 KB) of the DISCOVAR assembly (fig. 1*D*and*E*), and short-read data mapped to the genome had markedly lower depth in this region (fig. 1*B*), highlighting the difficulty of assembling and mapping highly repetitive regions with short reads.

We evaluated genome-wide synteny between PPM and two heteromyid species, Ord’s kangaroo rat (*Dipodomys ordii*) and banner-tailed kangaroo rat (*D. spectabilis*; Harder *et al*. 2022, fig. 1*D*and*E*). Although not closely related to PPM (22.3 Myr to the most recent common ancestor; Hafner *et al*. 2007), they are the closest species with contiguous reference genomes (scaffold N50 = 11.9 MB and contig N50 = 9.6 MB for *D. ordii* and *D. spectabilis*, respectively). Frequent rearrangements between genomes, as demonstrated by contigs in the *Dipodomys* genomes that map to multiple PPM chromosomes and vice versa, is consistent with high cytogenetic variability across rodents (Romanenko and Volobouev 2012; Zhou *et al*. 2020).

### Genome-Wide Heterozygosity and Demographic History

**Fig. 2.**
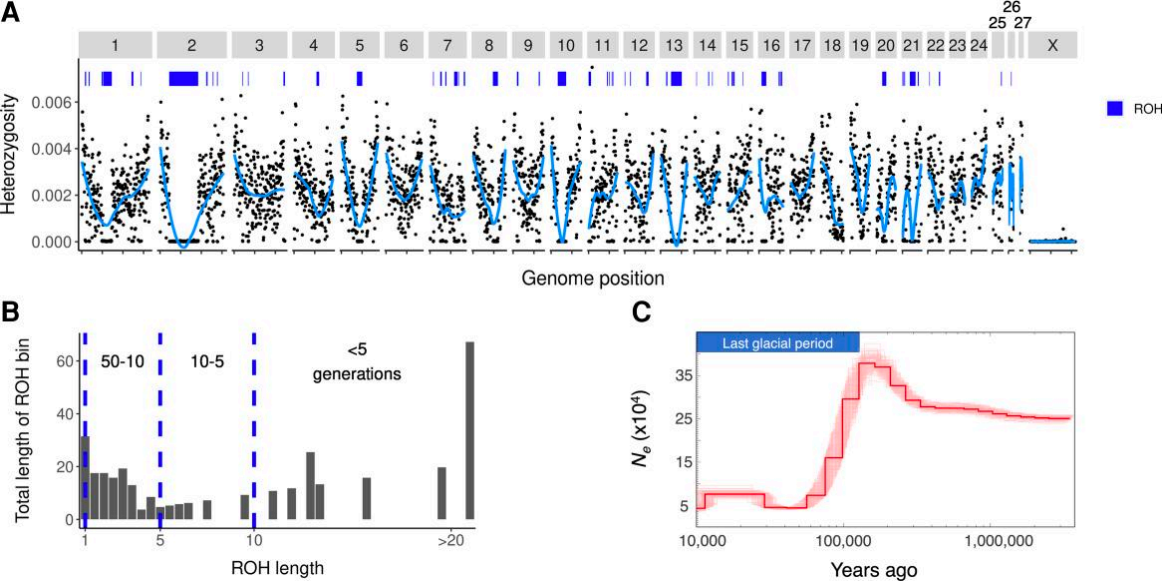
The distribution of heterozygosity across the PPM genome reflects recent inbreeding. (*A*) Mean heterozygosity in 500 KB windows (points), sliding mean heterozygosity (lines), and runs of homozygosity (ROH > 1 MB; bars at top). (*B*) Total lengths of ROH in each 1 MB bin (for 1 MB < ROH < 67 MB). Hatched lines and corresponding labels show the approximate number of generations since inbreeding for ROH of a given size range, assuming 100 MB tracts are inherited per meiosis. (*C*) *N_e_* over time, showing population decline at the beginning of the last glacial period followed by stable *N_e_* ∼ 50,000. Lighter lines are replicate PSMC runs, and the dark line shows the mean of all replicates.

The distribution of genetic diversity was highly variable across the genome. Heterozygosity was consistently elevated near telomeres, and heterozygous regions were interspersed with large spans of homozygosity. Mean autosomal heterozygosity was relatively high (2.00 × 10^−3^, ∼2 variants per KB), but 25.9% of autosomal 50 KB windows had virtually no heterozygosity (i.e., levels similar to those on the single-copy X chromosome) (fig. 2*A*). Nearly half (48.1%) of homozygous windows were part of ROH >5 MB in length, including a ∼67.1 MB span of chromosome 2 (fig. 2*A*and*B*). These long ROH likely stem from inbreeding within the last ten generations (fig. 2*B*), assuming 100 MB identity-by-descent tracts are inherited per meiosis (Thompson 2013; van der Valk *et al*. 2019). ROH are more common in regions of low recombination, for example on the X chromosome or within structural variants, but very long ROH are more likely to be caused by recent inbreeding (Ceballos *et al*. 2018).

In contrast with these signatures of contemporary inbreeding, Pairwise Sequentially Markovian Coalescent (PSMC) estimates suggest large historical effective population sizes for PPM. *N_e_* decreased between 150 and 100 Ka from ∼400,000 to ∼50,000, where it remained up to 10 Ka (fig. 2*C*). Selective sweeps, which are common in large populations, can also produce signals that appear as population contraction by PSMC (Schrider *et al*. 2016). The timing of the inferred historical population contraction coincides with the beginning of the last glacial period, a period of diversification, and speciation for many taxa in southern California (Vandergast *et al*. 2008), likely including *P. longimembris*, which consists of 16 recognized subspecies (www.itis.gov; accessed 13 December 2021). Other heteromyids show similar fluctuations and large historical *N_e_*, including the federally endangered Stephen’s kangaroo rat (*Dipodomys stephensi*; Harder *et al*. 2022). The large historical *N_e_* inferred from PSMC is supported by the relatively high heterozygosity outside of ROH windows (mean = 2.70 × 10^−3^).

The large historical *N_e_* stands in contrast with signatures of recent inbreeding in the reference individual. The ROH-based inbreeding coefficient, *F*_ROH > 1MB_ = 0.18, is on par with iconic endangered species such as South Asian tigers (*Panthera tigris*; Armstrong *et al*. 2021), some Iberian lynx (*Lynx pardinus*) populations (Abascal *et al*. 2016), Malayan pangolins (*Manis javanica*; Hu *et al*. 2020), and pygmy hogs (*Porcula salvania*; Liu *et al*. 2021). However, inbreeding can occur even when populations are large, especially for some heteromyid rodents with small natal dispersal distances (Waser *et al*. 2006), and thus inbreeding levels may vary across individuals. Heterozygosity at 15 previously published microsatellite loci suggests the reference individual was more inbred than its population, the largest of the three extant PPM populations (Spencer 2005). Heterozygosity was lower in the reference individual (proportion of heterozygous loci *pHt* = 0.47) than the population average (mean *pHt* = 0.70, *n* = 14; data from Wilder *et al*. 2020), but was not markedly different from PPM range-wide (mean *pHt* = 0.53, *n* = 35), including populations that have experienced the largest declines. Additional genomic samples are needed to estimate the level of inbreeding across the subspecies.

Habitat fragmentation from urbanization has led to population declines in this and other species across southern California (Bolger *et al*. 1997). Formerly large populations that recently contracted may be at increased risk of inbreeding depression from recessive deleterious variants, which may hinder recovery and exacerbate the threat of extinction (Hedrick and Garcia-Dorado 2016). This high-quality reference genome provides a critical tool for current and future studies of inbreeding depression, genetic load, structural variation, and outbreeding depression with population genomic data in this subspecies, and demonstrates the value of highly contiguous assemblies for the management and conservation of biodiversity.

## Materials and Methods

We provide an overview of the methods below. Further details can be found in the supplementary materials, Supplementary Material online.

### Sample Collection, DNA Extraction, and Library Preparation

A wild-born male PPM was collected in 2012 from Santa Margarita (San Diego, CA, USA), the largest and most genetically diverse extant population (Swei *et al*. 2003; Wilder *et al*. 2020). The individual was a founder for the PPM Conservation Breeding Program at the San Diego Zoo Wildlife Alliance (Shier 2014). A fibroblast cell line was established after the death of the individual in 2019 (King *et al*. 2020). Cells were harvested at passage 6 (for Omni-C data generated here and short-read data generated previously) and passage 11 (for RNAseq, HiFi, and Hi-C), cryopreserved and stored at −8°C.

### De Novo Genome Assembly

High molecular weight DNA was extracted from ∼20 M fibroblasts, and a Sequel HiFi Library was prepared and sequenced with two SMRT cells (Pacific Biosciences) using the Sequel II Sequencing Kit v2.0. We used the Peregrine assembler (Chin and Khalak) for the de novo assembly of 51.2 GB of HiFi reads with quality scores *Q* > 20 (23.3× genome coverage).

For the Dovetail Omni-C library, chromatin from ∼10.5 M fibroblasts was fixed in place with formaldehyde, extracted, and digested with DNAse I. Chromatin ends were repaired, adapter-containing ends were ligated by proximity ligation, crosslinks were reversed, and the DNA purified. Libraries were sequenced to ∼30× coverage. The HiFi-based draft and Omni-C library reads with MQ > 50 were then assembled using HiRise (Putnam *et al*. 2016).

The HiFi + Omni-C assembly was then scaffolded to chromosome-length by the DNA Zoo Consortium following the methodology described here: www.dnazoo.org/methods. Briefly, in situ Hi-C data from ∼2 M fibroblasts were generated following the Rao *et al*. (2014) protocol, processed using Juicer (Durand, Shamim, *et al*. 2016), and input into the 3D-DNA pipeline (Dudchenko *et al*. 2017) to produce a candidate chromosome-length genome assembly. We performed additional finishing on the scaffolds using Juicebox Assembly Tools (Durand, Robinson, *et al*. 2016; Dudchenko *et al*. 2018).

We assembled the mitogenome using both the HiFi data and publicly available short-read data (SRR11431899) from the same individual. We assembled mitogenomes from the short-read data using NOVOPlasty (Dierckxsens *et al*. 2020) and GetOrganelle (Jin *et al*. 2020), then mapped HiFi reads to the consensus using minimap2 (Li 2021), retaining primary reads <16,500-bp, to generate a consensus mitogenome sequence.

We summarized statistics of the assemblies using the assemblathon_stats.pl script, and assessed the completeness of the assembly against the eukaryota_odb10 and glires_odb10 databases using BUSCO, ver 4.0.1 (Simão *et al*. 2015). To estimate the genome size, we quantified k-mers (17–25-mer) from the short-read data using Jellyfish (Marçais and Kingsford 2011) and GenomeScope (Vurture *et al*. 2017).

We evaluated synteny between the PPM genome assembly and the Ord’s kangaroo rat (*D. ordii*) genome (Dord_2.0) and banner-tailed kangaroo rat (*D. spectabilis*) genome (GCA_019054845.1). We aligned chromosome-length PPM scaffolds and scaffolds >10 MB for each kangaroo rat using lastz v1.04 (Harris 2007). We compared PPM genome assemblies by liftOver using Flo (https://github.com/wurmlab/flo).

### Gene Annotation

Genes were annotated by the NCBI Eukaryotic Genome Annotation Pipeline v. 9.0 (Thibaud-Nissen et al. 2013). RNA was extracted from fibroblasts, liver, skeletal muscle, and heart (supplemental table S4, Supplementary Material online). Libraries were generated from 100 ng of total RNA, and 9.96 GB of 75-bp reads were used for gene prediction, along with RNAseq data from kidney and spleen tissue of four Bailey's pocket mouse (*Chaetodipus baileyi*) samples (SAMN03068786-9), and from five kidney samples of rock pocket mice (*Chaetodipus intermedius*; SAMN15773208-12). The mitogenome was annotated with the MITOS Web server (Bernt *et al*. 2013) and visualized with MitoAnnotator (Iwasaki *et al*. 2013).

### Heterozygosity, ROH, and PSMC Analysis

We estimated variant sites across the genome using the publicly available, paired-end 250 bp read data (SRR11431899). We trimmed adapters, mapped the reads to our assembly, removed read duplicates, and called variants using HaplotypeCaller in GATK v3.8 (Van der Auwera and O’Connor 2020). We estimated heterozygosity in 50–500 KB windows, accounting for sites with missing data using pixy v.1.1 (Korunes and Samuk 2021). To estimate ROH, we summed consecutive 50 KB windows with heterozygosity < 0.0002 (0.2 variants/KB). This threshold was equivalent to the 99th percentile of the heterozygosity distribution on the X chromosome, which is haploid in the male reference individual and thus represents sequencing and mapping error. To prevent long ROH from being spuriously broken, single 50 KB windows with heterozygosity < 0.0004 that had mean heterozygosity < 0.0002 in the surrounding 1 MB were allowed in ROH.

To estimate historical trends in effective population size, we ran 25 replicates of PSMC (Li and Durbin 2011), using settings established for the Norway rat (Deinum *et al*. 2015), a mutation rate of 2.96e−09 and generation time of 1 year.

## Supplementary Material

evac122_Supplementary_Data

## Data Availability

The contact matrices are available at https://www.dnazoo.org/assemblies/Perognathus_longimembris_pacificus and https://tinyurl.com/25vywofz. The genome assembly has been deposited at DDBJ/ENA/GenBank under the accession JALGBO000000000. Sequence data have been deposited at the NCBI under PRJNA818714. The gene annotation is available as NCBI *Perognathus longimembris pacificus* Annotation Release 100.
