# Supplementary material for: A Chromosome-Length Reference Genome for the Endangered Pacific Pocket Mouse Reveals Recent Inbreeding in a Historically Large Population
Source: Genome Biol Evol. 2022 Jul 27;14(8):evac122. doi: 10.1093/gbe/evac122 (PMC9348616; doi:10.1093/gbe/evac122)
Supplement: evac122_Supplementary_Data [file evac122_supplementary_data.docx]

Supplemental Materials

A chromosome-length reference genome for the endangered Pacific pocket mouse reveals recent inbreeding in a historically large population

Aryn P. Wilder, Olga Dudchenko, Caitlin Curry, Marisa Korody, Sheela P. Turbek, Mark Daly, Ann Misuraca, Gaojianyong WANG, Ruqayya Khan, David Weisz, Julie Fronczek, Erez Lieberman Aiden, Marlys L. Houck, Debra M. Shier, Oliver A. Ryder, Cynthia C. Steiner

## Fibroblast cell culture

Fibroblast cell lines were established from post mortem ear and trachea samples and accessioned into the San Diego Zoo Wildlife Alliance’s Frozen Zoo® (Lab # KB21356). Aliquots of fibroblasts were harvested at passage 6 and frozen at -80C in 1X PBS with 10% glycerol and 10% DMSO. Aliquots harvested at passage 11 were cryopreserved in DMEM with 10% FBS, slow-cooled in a CoolCell and stored at -80C. Passage 6 samples were used for generating Omni-C and Illumina short-read data (SRR11431899), whereas passage 11 samples were used for HiFi, Hi-C and RNAseq data generation.

Metaphase chromosomes of both tissues were examined by non-differential staining using Giemsa; the ear tissue was additionally G- and C-banded. Cell culturing, harvesting, and chromosome banding followed the techniques described by Kumamoto et al. (1996). Karyotyping was done using the CytoVision Genus® system by Leica Microsystems.

##

## Karyotyping

The diploid number for the passage 6 fibroblast sample was 2n=57-58,XY. Karyotypes from other PPM individuals typically range from 2n=56-58, with 2n=56 being most common and consistent with the ancestral diploid number inferred by McKnight (1995). 13 of the 35 cells counted had 57 chromosomes, suggesting some artifacts resulting from fibroblast culture that are typical of cells that are difficult to culture, and potentially explaining the disagreement between the karyotype-based diploid number for this individual and the number of chromosome-length scaffolds. The passage 11 sample had high levels of polyploidy, with most cells showing tetraploidy and above. Given that polyploidy arose between passages 6 and 11, it likely stems from chromosomal duplication events yielding largely identical sequences. Recent duplication would affect the evenness of sequence read coverage, but would likely not generate additional haplotypes. Small differences between copies may be output as additional contigs (haplotigs or primary contigs), but these would likely be collapsed in downstream scaffolding steps.

## High molecular weight DNA extraction

DNA was extracted from ~20M fibroblast cells using the QIAGEN Genomic-tip 100/G (QIAGEN, Hilden, Germany) following manufacturer's instructions. Briefly, cells were washed twice and resuspended in PBS, and lysed in cold Buffer C1. The lysed cells were centrifuged and the supernatant discarded, and then washed with cold Buffer C1 to remove the cell debris from the nuclear pellet. The pellet was then resuspended in Buffer G2 and incubated with Proteinase K to strip the genomic DNA of bound proteins. The sample lysate was applied to the equilibrated QIAGEN Genomic-tip, washed, eluted and precipitated with isopropanol. The sample was immediately mixed and centrifuged and the DNA pellet was washed with cold 70% ethanol, vortexed and centrifuged again. The supernatant was removed and the pellet was air dried, resuspended in Buffer EB.

## Sequel HiFi Library Construction and Sequel Ⅱ Sequencing

The Sequel HiFi Library was prepared according to the manufacturer’s protocol. Briefly, 15ug sample was sheared step using Diagenode’s Megaruptor system. A SMRTbell library was constructed by using SMRTbell® Express Template Preparation Kit v2. We measured the concentration and size of the library with the Qubit dsDNA HS Assay, Agilent 12000 DNA kit, respectively, and removed small fragments with BluePippin. The sequencing primer v4 was annealed to the SMRTbell template, DNA polymerase was bound to the complex (Sequel Ⅱ Binding kit 2.0). Excess primer and polymerase was removed using AMPure beads prior to sequencing. The library was sequenced with 2 SMRT cells (Pacific Biosciences) using the Sequel Ⅱ Sequencing Kit v2.0. 1 × 34 hour movies (30hr movie time, 4hr pre extension time) were captured for each SMRT Cell 8M on the Sequel Ⅱ (Pacific Biosciences) sequencing platform, and bases were called using SMRTLink v8.0.

##

## De novo genome assembly and scaffolding

A total of 65.41 GB of raw PacBio read data were generated, comprising 51.23 GB of HiFi read data across 2.25 M reads that passed >Q20 filter (99%), with N50 read length of 22.2 KB (Table S1; Figure S1). The *de novo* Peregrine assembly of HiFi reads had 7,031 contigs ranging from 19.8 KB to 36.8 MB and a contig N50 of 8.47 MB.

For each Dovetail Omni-C library, chromatin from ~10.5M fibroblast cells was fixed in place with formaldehyde in the nucleus and then extracted. Fixed chromatin was digested with DNAse I, chromatin ends were repaired and ligated to a biotinylated bridge adapter followed by proximity ligation of adapter containing ends. After proximity ligation, crosslinks were reversed and the DNA purified. Purified DNA was treated to remove biotin that was not internal to ligated fragments. Sequencing libraries were generated using NEBNext Ultra enzymes and Illumina-compatible adapters. Biotin-containing fragments were isolated using streptavidin beads before PCR enrichment of each library. The library was sequenced on an Illumina HiSeqX platform to produce approximately 30x sequence coverage. Then HiRise used MQ>50 reads for scaffolding. The HiFi-based de novo assembly and Omni-C library reads were used as input data for HiRise (Putnam et al. 2016). Omni-C library sequences were aligned to the draft input assembly using bwa (https://github.com/lh3/bwa). The separations of Omni-C read pairs mapped within draft scaffolds were analyzed by HiRise to produce a likelihood model for genomic distance between read pairs, and the model was used to identify and break putative misjoins, to score prospective joins, and make joins above a threshold. 188,980,543 read pairs of Omni-C data were generated, resulting in 266 joins and 796 breaks in the input assembly.

The HiFi+Omni-C assembly was then scaffolded to chromosome-length by the DNA Zoo Consortium following the methodology described here: www.dnazoo.org/methods. Briefly, Hi-C libraries were prepared from ~2M fibroblast cells. In situ Hi-C data generated following the Rao et al. (2014) protocol was processed using Juicer (Durand, Shamim, et al. 2016), and used as input into the 3D-DNA pipeline (Dudchenko et al. 2017) to produce a candidate chromosome-length genome assembly. We performed additional finishing on the scaffolds using Juicebox Assembly Tools (Durand, Robinson, et al. 2016; Dudchenko et al. 2018) to produce the final assembly (Table S2). The contact matrices generated by aligning the Hi-C data to the genome assembly before and after the Hi-C scaffolding are available for browsing at multiple resolutions on https://www.dnazoo.org/assemblies/Perognathus_longimembris_pacificus visualized using Juicebox.js, a cloud-based visualization system for Hi-C data (Robinson et al. 2018).

## Mitogenome assembly

We assembled the mitogenome from both the short-read data (SRR11431899) and HiFi data generated here from the same individual. First, we assembled *de novo* mitogenomes from the short-read data using NOVOPlasty (Dierckxsens et al. 2020) and GetOrganelle (Jin et al. 2020), and aligned the two to create a consensus sequence. We then mapped HiFi reads to the consensus using minimap2 (Li 2021), retaining primary reads <16,500-bp with <5% divergence from the consensus (23.6X coverage). We identified variants using mpileup and the multiallelic caller in bcftools (Li et al. 2019), with MAPQ≥40 and base QV≥30, and called the consensus haplotype. The mitogenome was annotated with the MITOS Web server (Bernt et al. 2013) and visualized with MitoAnnotator (Iwasaki et al. 2013; Figure S2 and Table S3).

## RNA extraction, library preparation and sequencing

RNA was extracted from four tissues: passage 11 fibroblasts, liver stored in RNA*later* (Invitrogen), skeletal muscle with attached skin, and heart (Table S4). Fibroblasts were removed from the flask using 0.05% Trypsin – EDTA, pelleted at 0.3 g for five minutes, washed once in DPBS, resuspended in 600 mL of lysis buffer from the *mir*Vana™ miRNA Isolation kit (Invitrogen) and stored at -80°C until extraction. Tissue was collected and stored at -80 °C until use when it was finely minced on dry ice using a scalpel in the presence of lysis buffer. RNA was then extracted from the cells or tissue following the manufacturer’s instructions for a total RNA fraction. RNA was quantified using the Qubit Fluorometer (Thermo Fisher Scientific) and 100ng of total RNA was then used for the library preparation. RIN scores were obtained from a TapeStation 4150 (Agilent, Santa Clara CA) using the High Sensitivity RNA Screentape Assay.

Total RNA was Poly(A) selected using the NEBNext® Poly(A) mRNA Magnetic Isolation Module as part of the library generation protocol for the NEBNext® Ultra™ II Directional RNA Library Prep Kit for Illumina® (New England Biolabs, Ipswich, MA) following the manufacturer’s instructions. RNA fragmentation was chemically performed at 94°C for 15 minutes for a target insert size of 200 bp and the PCR enrichment step performed for 12 cycles. Library concentrations were determined using Qubit and insert sizes were obtained using the DNA High Sensitivity Bioanalyzer Assay (Agilent Technologies) and diluted for sequencing. Sequencing was performed on an Illumina NextSeq 500 with 76 bp paired end reads by the Sanford Burnham Prebys Genomics Core (La Jolla, CA).

## Annotation

The HiFi assembly contained 96.4% complete BUSCOs from the Eukaryota database (eukaryota_odb10), and 92.3% complete BUSCO’s from the Glires database (​​glires_odb10). RepeatMasker showed a total GC content of 41.98%, with 13.64% of the genome categorized as SINEs, 10.46% as LINEs, and 38.67% of the genome as total interspersed repeats (Table S5).

Genes were annotated by the NCBI Eukaryotic Genome Annotation Pipeline v. 9.0 (Thibaud-Nissen et al. 2013; for details, see https://www.ncbi.nlm.nih.gov/genome/annotation_euk/process/). Gene prediction included RNAseq data from three PPM tissues generated as described above, as well as RNAseq from kidney and spleen tissue of four Bailey's pocket mouse (*Chaetodipus baileyi*) individuals (SAMN03068786, SAMN03068787, SAMN03068788, SAMN03068789), and from kidney of five rock pocket mice (*Chaetodipus intermedius*; SAMN15773208, SAMN15773209, SAMN15773210, SAMN15773211, SAMN15773212).

## Comparing genome assemblies

We evaluated synteny between the PPM genome assembly and the Ord’s kangaroo rat (*Dipodomys ordii*) genome (Dord_2.0) and banner-tailed kangaroo rat (*D. spectabilis*) genome (GCA_019054845.1). We aligned the 28 chromosome-length scaffolds for PPM and scaffolds > 10MB for each kangaroo rat using lastz v1.04, ignoring gapped alignments and lowering seed sensitivity to reduce runtime (‑‑nogapped ‑‑notransition --step=20) (Harris 2007). We compared our new genome assembly to the previous draft assembly of the PPM genome by generating liftover chain files between assemblies using Flo (https://github.com/wurmlab/flo). We ran the program under default settings, except we used -fastMap -tileSize=12 -minIdentity=98. We visualized syntenic relationships between the two heteromyid species for alignments >500bp in length with Circos (Krzywinski et al. 2009), and plotted alignments between PPM genome versions that were >100KB in length.

## Short read mapping, kmer analysis, and heterozygosity estimation

We downloaded paired-end 250 bp read data (SRR11431899) to estimate variant sites across the genome. We trimmed TruSeq adapters with Trimmomatic v.0.39 (Sewe et al. 2022), mapped the short-read data to our assembly using the very-sensitive-local setting in bowtie2 (Langmead & Salzberg 2012), and removed PCR duplicates using sambamba 0.7.1 (Tarasov et al. 2015). Depth and quality score distributions of mapped reads were assessed with bcftools stats (Li et al. 2019) and mosdepth (Pedersen & Quinlan 2018). The overall mapping rate was high (97.46%), with 68.38% of read pairs mapping concordantly and uniquely, and 27.05% of reads mapping concordantly to multiple regions of the genome. 3.95% of total reads were PCR duplicates and removed, and the final reads had a mean depth of 35.8x coverage of the genome. We called variants using HaplotypeCaller in Genome Analysis Toolkit (GATK) v3.8 (Van der Auwera & O’Connor 2020), emitting all sites (variant and invariant) with mapping quality ≥20, and depth ≥10 and ≤62 reads and a quality score ≥20. A total of 5.98 M sites were heterozygous across the genome of the reference individual.

We used Jellyfish v.1.1.4 (Marçais & Kingsford 2011) and GenomeScope (Vurture et al. 2017) to estimate the genome size from kmers (17mer, 19mer, 21mer and 25mer). Model fit was highest under k=25 (96.0-97.6%) and the haploid genome length was estimated to be 1.83GB (Figure S3; Table S6).

# References

Bernt M et al. 2013. MITOS: improved de novo metazoan mitochondrial genome annotation. Mol. Phylogenet. Evol. 69:313–319.

Bradnam KR et al. 2013. Assemblathon 2: evaluating de novo methods of genome assembly in three vertebrate species. Gigascience. 2:10.

Brůna T, Hoff KJ, Lomsadze A, Stanke M, Borodovsky M. 2021. BRAKER2: automatic eukaryotic genome annotation with GeneMark-EP+ and AUGUSTUS supported by a protein database. NAR Genom Bioinform. 3. doi: 10.1093/nargab/lqaa108.

Caballero M, Wegrzyn J. 2019. gFACs: Gene Filtering, Analysis, and Conversion to Unify Genome Annotations Across Alignment and Gene Prediction Frameworks. Genomics Proteomics Bioinformatics. 17:305–310.

Dierckxsens N, Mardulyn P, Smits G. 2020. Unraveling heteroplasmy patterns with NOVOPlasty. NAR Genomics and Bioinformatics. 2. doi: 10.1093/nargab/lqz011.

Dobin A et al. 2013. STAR: ultrafast universal RNA-seq aligner. Bioinformatics. 29:15–21.

Dudchenko O et al. 2017. De novo assembly of the genome using Hi-C yields chromosome-length scaffolds. Science. 356:92–95.

Dudchenko O et al. 2018. The Juicebox Assembly Tools module facilitates de novo assembly of mammalian genomes with chromosome-length scaffolds for under $1000. bioRxiv. 254797. doi: 10.1101/254797.

Durand NC, Robinson JT, et al. 2016. Juicebox Provides a Visualization System for Hi-C Contact Maps with Unlimited Zoom. Cell Syst. 3:99–101.

Durand NC, Shamim MS, et al. 2016. Juicer Provides a One-Click System for Analyzing Loop-Resolution Hi-C Experiments. Cell Syst. 3:95–98.

Harris RS. 2007. *Improved Pairwise Alignment of Genomic DNA*.

Hoff KJ, Lange S, Lomsadze A, Borodovsky M, Stanke M. 2016. BRAKER1: Unsupervised RNA-Seq-Based Genome Annotation with GeneMark-ET and AUGUSTUS. Bioinformatics. 32:767–769.

Iwasaki W et al. 2013. MitoFish and MitoAnnotator: a mitochondrial genome database of fish with an accurate and automatic annotation pipeline. Mol. Biol. Evol. 30:2531–2540.

Jin J-J et al. 2020. GetOrganelle: a fast and versatile toolkit for accurate de novo assembly of organelle genomes. Genome Biol. 21:241.

Krzywinski M et al. 2009. Circos: an information aesthetic for comparative genomics. Genome Res. 19:1639–1645.

Kumamoto AT, Charter SJ, Houck ML, Frahm M. 1996. Chromosomes of Damaliscus (Artiodactyla, Bovidae): simple and complex centric fusion rearrangements. Chromosome Res. 4:614–621.

Langmead B, Salzberg SL. 2012. Fast gapped-read alignment with Bowtie 2. Nat. Methods. 9:357–359.

Li H. 2021. New strategies to improve minimap2 alignment accuracy. Bioinformatics. doi: 10.1093/bioinformatics/btab705.

Li H, Handsaker B, Danecek P, McCarthy S, Marshall J. 2019. *BCFtools*.

Marçais G, Kingsford C. 2011. A fast, lock-free approach for efficient parallel counting of occurrences of k-mers. Bioinformatics. 27:764–770.

McKnight ML. 1995. Mitochondrial DNA Phylogeography of Perognathus amplus and Perognathus longimembris (Rodentia: Heteromyidae): a possible mammalian ring species. Evolution. 49:816–826.

Pedersen BS, Quinlan AR. 2018. Mosdepth: quick coverage calculation for genomes and exomes. Bioinformatics. 34:867–868.

Putnam NH et al. 2016. Chromosome-scale shotgun assembly using an in vitro method for long-range linkage. Genome Res. 26:342–350.

Rao SSP et al. 2014. A 3D map of the human genome at kilobase resolution reveals principles of chromatin looping. Cell. 159:1665–1680.

Robinson JT et al. 2018. Juicebox.js Provides a Cloud-Based Visualization System for Hi-C Data. Cell Syst. 6:256–258.e1.

Sewe SO, Silva G, Sicat P, Seal SE, Visendi P. 2022. Trimming and Validation of Illumina Short Reads Using Trimmomatic, Trinity Assembly, and Assessment of RNA-Seq Data. Methods Mol. Biol. 2443:211–232.

Tarasov A, Vilella AJ, Cuppen E, Nijman IJ, Prins P. 2015. Sambamba: fast processing of NGS alignment formats. Bioinformatics. 31:2032–2034.

Thibaud-Nissen F, Souvorov A, Murphy T, DiCuccio M, Kitts P.2013. Eukaryotic genome annotation pipeline. In: The NCBI handbook [Internet]. 2nd ed. Bethesda (MD): National Center for Biotechnology Information.

Van der Auwera GA, O’Connor BD. 2020. *Genomics in the Cloud: Using Docker, GATK, and WDL in Terra*. O’Reilly Media.

Vurture GW et al. 2017. GenomeScope: fast reference-free genome profiling from short reads. Bioinformatics. 33:2202–2204.

#

# **Supplemental Figures and Tables**


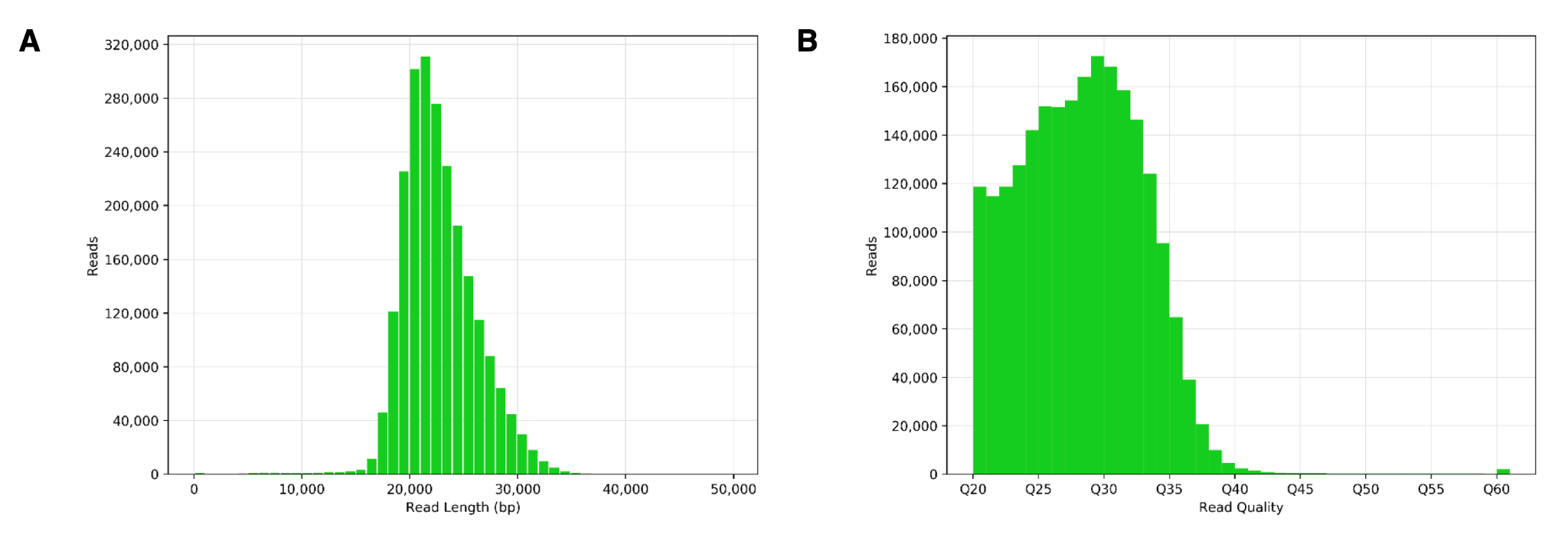


### Figure S1. Read length and read quality distributions for 2.3 M PacBio HiFi reads (51.2 GB) used for the *de novo* assembly. N50 read length = 22.7 KB and maximum read length = 49.8 KB.


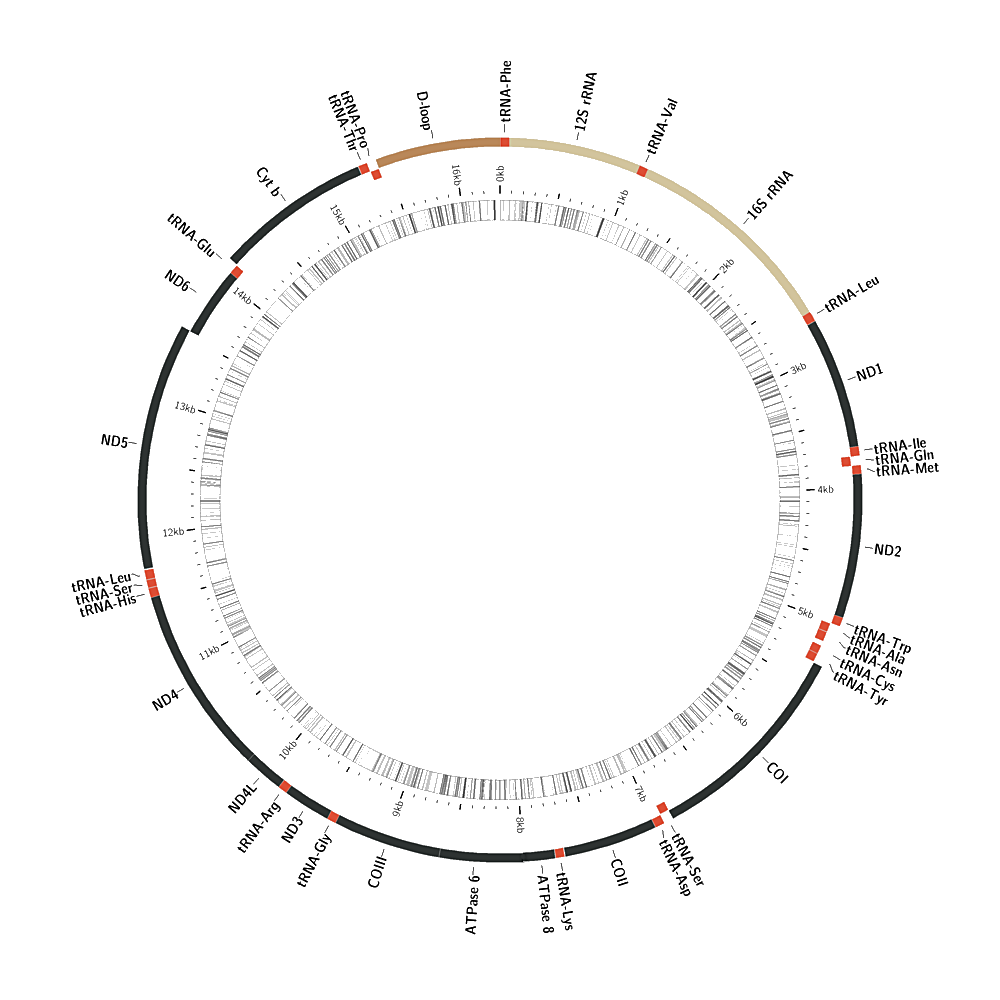


### Figure S2. Visualization of the mitogenome via MitoAnnotator (doi: 10.1093/molbev/mst141) showing placement of genes and their locations on the H and L strands. The inner circle shows GC% per 5bp of the mitogenome with darker lines representing higher GC%.

###


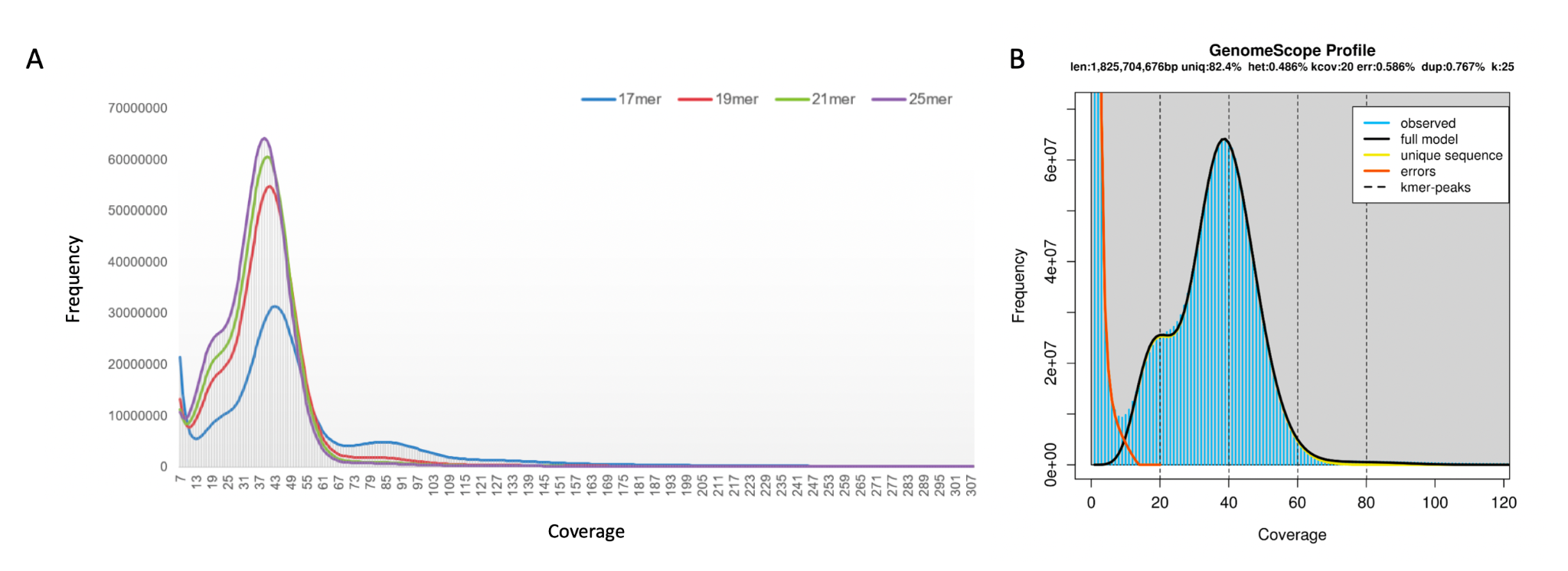


### Figure S3. A) Distribution of kmers in the Illumina short-read data output by Jellyfish for 17mer, 19mer, 21mer and 25mer. B) GenomeScope profile for k=25, estimating genome length of 1.83GB.

###

| Table S1. Summary of sequencing data generated by two SMRT cells of Pacific Biosciences Sequel II. | | | | | | |
| --- | --- | --- | --- | --- | --- | --- |
|  | **Number of bases** | **Number of reads** | **N50 read length** | **Mean read length** | **Mean insert length** | **Max read length** |
| Raw reads | 654,053,095,409 | 7,406,779 | 183,250 | 88,305 | 21,004 | - |
| Subreads | 654,815,845,773 | 34,531,273 | 22,233 | 18,963 | - | 420,472 |
| HiFi reads | 51,227,145,714 | 2,253,708 | 22,726 | 22,730 | - | 49,819 |

###

###

| Table S2. Genome assembly statistics comparing the final assembly (HiFi + Omni-C + Hi-C) to earlier iterations (HiFi + Omni-C assembly and HiFi only assembly), and to the draft DISCOVAR assembly. | | | | |
| --- | --- | --- | --- | --- |
|  | **Final assembly (HiFi + Omni-C + Hi-C)** | **HiFi + Omni-C** | **HiFi** | **Discovar** |
| Number of scaffolds | 6,180 | 7,534 | 7,031 | 2,409,818 |
| Total size of scaffolds | 2,212,099,196 | 2,211,328,296 | 2,211,301,696 | 2,601,695,796 |
| Longest scaffold | 163,161,067 | 86,151,820 | 36,783,186 | 625,731 |
| Shortest scaffold | 919 | 1,280 | 19,812 | 200 |
| Number of scaffolds > 1K nt | 6,176 (99.9%) | 7,534 (100%) | 7,031 (100%) | 167,590 (7.0%) |
| Number of scaffolds > 10K nt | 6,066 (98.2%) | 7,489 (99.4%) | 7,031 (100%) | 43,109 (1.8%) |
| Number of scaffolds > 100K nt | 288 (4.7%) | 848 (11.3%) | 903 (12.8%) | 2,314 (0.1%) |
| Number of scaffolds > 1M nt | 31 (0.5%) | 80 (1.1%) | 278 (4.0%) | 0 (0%) |
| Number of scaffolds > 10M nt | 27 (0.4%) | 48 (0.6%) | 59 (0.8%) | 0 (0%) |
| Mean scaffold size | 357,945 | 293,513 | 314,507 | 1,080 |
| Median scaffold size | 38,905 | 40,641 | 40,997 | 227 |
| N50 scaffold length | 72,679,016 | 35,363,251 | 8,466,147 | 24,714 |
| L50 scaffold count | 11 | 23 | 70 | 23,202 |
| scaffold %A | 29 | 29 | 29.03 | 29.04 |
| scaffold %C | 20.98 | 20.98 | 20.99 | 20.95 |
| scaffold %G | 20.98 | 20.99 | 20.99 | 20.89 |
| scaffold %T | 29 | 29.02 | 29 | 29.05 |
| scaffold %N | 0.04 | 0 | 0 | 0.07 |
| scaffold %non-ACGTN | 0 | 0 | 0 | 0 |
| Number of scaffold non-ACGTN nt | 0 | 0 | 0 | 0 |
|  |  |  |  |  |
| Percentage of assembly in scaffolded contigs | 87.9% | 74.4% | 0.0% | 30.6% |
| Percentage of assembly in unscaffolded contigs | 12.1% | 25.6% | 100% | 69.4% |
| Average number of contigs per scaffold | 1.3 | 1 | 1 | 1 |
| Average length of break (>25 Ns) between contigs in scaffold | 441 | 100 | 0 | 100 |
|  |  |  |  |  |
| Number of contigs | 7,987 | 7,800 | 7,031 | 2,428,619 |
| Number of contigs in scaffolds | 1,933 | 313 | 0 | 31,529 |
| Number of contigs not in scaffolds | 6,054 | 7,487 | 7,031 | 2,397,090 |
| Total size of contigs | 2,211,301,696 | 2,211,301,696 | 2,211,301,696 | 2,599,815,696 |
| Longest contig | 36,783,186 | 36,783,186 | 36,783,186 | 338,096 |
| Shortest contig | 4 | 1,280 | 19,812 | 200 |
| Number of contigs > 1K nt | 7,938 (99.4%) | 7,800 (100%) | 7,031 (100%) | 186,391 (7.7%) |
| Number of contigs > 10K nt | 7,796 (97.6%) | 7,755 (99.4%) | 7,031 (100%) | 54,658 (2.3%) |
| Number of contigs > 100K nt | 1,118 (14.0%) | 1,112 (14.3%) | 903 (12.8%) | 751 (0.0%) |
| Number of contigs > 1M nt | 273 (3.4%) | 272 (3.5%) | 278 (4.0%) | 0 (0%) |
| Number of contigs > 10M nt | 55 (0.7%) | 56 (0.7%) | 59 (0.8%) | 0 (0%) |
| Mean contig size | 276,863 | 283,500 | 314,507 | 1,070 |
| Median contig size | 40,897 | 41,327 | 40,997 | 227 |
| N50 contig length | 7,389,774 | 7,389,774 | 8,466,147 | 17,686 |
| L50 contig count | 73 | 73 | 70 | 34,664 |
| contig %A | 29.02 | 29 | 29.03 | 29.06 |
| contig %C | 20.99 | 20.98 | 20.99 | 20.97 |
| contig %G | 20.99 | 20.99 | 20.99 | 20.9 |
| contig %T | 29.01 | 29.02 | 29 | 29.07 |
| contig %N | 0 | 0 | 0 | 0 |
|  |  |  |  |  |
| contig %non-ACGTN | 0 | 0 | 0 | 0 |
| Number of contig non-ACGTN nt | 0 | 0 | 0 | 0 |

###

###

| Table S3. Mitogenome genes, locations in the reference, strand and gene length. | | | | |
| --- | --- | --- | --- | --- |
| **Gene name** | **Start** | **Stop** | **Strand** | **Length** |
| cox2-0 | 2 | 448 | + | 447 |
| trnK(aaa) | 456 | 520 | + | 65 |
| atp8 | 523 | 711 | + | 189 |
| atp6 | 684 | 1358 | + | 675 |
| cox3 | 1364 | 2146 | + | 783 |
| trnG(gga) | 2148 | 2215 | + | 68 |
| nad3 | 2216 | 2560 | + | 345 |
| trnR(cga) | 2563 | 2629 | + | 67 |
| nad4l | 2647 | 2925 | + | 279 |
| nad4 | 2922 | 4286 | + | 1365 |
| trnH(cac) | 4297 | 4366 | + | 70 |
| trnS1(agc) | 4368 | 4426 | + | 59 |
| trnL1(cta) | 4430 | 4499 | + | 70 |
| nad5 | 4507 | 6297 | + | 1791 |
| nad6 | 6309 | 6827 | - | 519 |
| trnE(gaa) | 6828 | 6898 | - | 71 |
| cob | 6904 | 8037 | + | 1134 |
| trnT(aca) | 8046 | 8112 | + | 67 |
| trnP(cca) | 8114 | 8179 | - | 66 |
| Dloop | 8180 | 9048 |  |  |
| trnF(ttc) | 9049 | 9117 | + | 69 |
| rrnS | 9118 | 10076 | + | 959 |
| trnV(gta) | 10077 | 10143 | + | 67 |
| rrnL | 10142 | 11713 | + | 1572 |
| trnL2(tta) | 11714 | 11787 | + | 74 |
| nad1 | 11790 | 12737 | + | 948 |
| trnI(atc) | 12745 | 12813 | + | 69 |
| trnQ(caa) | 12811 | 12881 | - | 71 |
| trnM(atg) | 12881 | 12949 | + | 69 |
| nad2 | 12950 | 13987 | + | 1038 |
| trnW(tga) | 13992 | 14057 | + | 66 |
| trnA(gca) | 14062 | 14129 | - | 68 |
| trnN(aac) | 14131 | 14203 | - | 73 |
| trnC(tgc) | 14237 | 14303 | - | 67 |
| trnY(tac) | 14305 | 14369 | - | 65 |
| cox1 | 14375 | 15910 | + | 1536 |
| trnS2(tca) | 15920 | 15989 | - | 70 |
| trnD(gac) | 15993 | 16062 | + | 70 |
| cox2-1 | 16171 | 16266 | + | 96 |

###

###

###

| Table S4. Pacific Pocket Mouse tissue samples used for RNA extraction and gene annotation, including studbook (i.e. pedigree), lab ID number, sex, sample type, and RIN score of the RNA extraction. | | | | |
| --- | --- | --- | --- | --- |
| **Lab ID Number** | **Studbook Number** | **Sex** | **Sample Type** | **RIN** |
| KB 21356 (reference individual) | 17 | M | Passage 11 fibroblasts | 9 |
| OR 8263 | 30 | F | Liver stored in RNAlater | 1.6 |
| OR 7968 | 109 | F | Skeletal muscle with skin | 2.9 |
| OR 5310 | 10 | M | Heart | 7.3 |

###

| Table S5. Repeat content in the assembly predicted from RepeatMasker, showing the repeat type, number of elements, total length and percentage of the genome represented by each type. In total, 41% of the genome was classified as repetitive. | | | |
| --- | --- | --- | --- |
| **Repeat** | **Number of Elements** | **Length Occupied (bp)** | **Percentage of Sequence (%)** |
| SINEs | 1,799,014 | 301,626,535 | 13.64 |
| LINEs | 709,384 | 231,368,337 | 10.46 |
| LTR elements | 297,077 | 186,184,730 | 8.42 |
| DNA elements | 130,859 | 30,788,604 | 1.39 |
| Unclassified | 175,026 | 105,117,219 | 4.75 |
| Total interspersed repeats |  | 855,085,425 | 38.67 |
| Small RNA | 1,436 | 521,211 | 0.02 |
| Satellites | 50,999 | 6,725,431 | 0.30 |
| Simple repeats | 789,473 | 48,638,706 | 2.20 |
| Low complexity | 103,035 | 6,859,178 | 0.31 |

###

| Table S6. K-mer analysis of genome size and repeat length based on Illumina short-read data. (Where minimum and maximum predicted values differ at the level of significant digits, two numbers are shown.) | | | |
| --- | --- | --- | --- |
|  | **19mer** | **21mer** | **25mer** |
| Total nucleotides | 77,461,693,321 | 76,609,612,667 | 75,585,973,482 |
| Peak depth | 41 | 40 | 39 |
| Predicted genome size (Jellyfish) | 1,889,309,593 | 1,915,240,317 | 1,938,101,884 |
| Heterozygosity | 0.515-0.517% | 0.513-0.515% | 0.485-0.487% |
| Haploid length | 1.776 GB | 1.795-1.796 GB | 1.825-1.826 GB |
| Repeat length | 476.4-476.6 MB | 365.6-365.8 MB | 320.5-320.7 MB |
| Unique length | 1.299-1.300 GB | 1.430 GB | 1.504-1.505 GB |
| Model fit | 95.32-97.70% | 95.69-97.57% | 96.00-97.58% |
| Read error rate | 0.58% | 0.61% | 0.59% |

### 
